# CHRDL2 activates the PI3K/AKT pathway to ameliorate glucocorticoid-induced damages to bone microvascular endothelial cells (BMECs)

**DOI:** 10.1016/j.heliyon.2024.e33867

**Published:** 2024-06-28

**Authors:** Xianzhe Huang, Shuo Jie, Wenzhao Li, Chan Liu

**Affiliations:** a Department of Orthopedics, The Second Xiangya Hospital, Central South University, Changsha, Hunan, 410011, China; b International Medical Department, The Second Xiangya Hospital, Central South University, Changsha, Hunan, 410011, China

**Keywords:** Avascular necrosis of the femoral head (ANFH), Bone microvascular endothelial cells (BMECs), Glucocorticoids (GCs), Chordin-like 2 (CHRDL2)

## Abstract

Steroid-induced avascular necrosis of the femoral head (ANFH) is characterized by the death of bone tissues, leading to the impairment of normal reparative processes within micro-fractures in the femoral head. Glucocorticoid (GCs)-induced bone microvascular endothelial cell (BMEC) damage has been reported to contribute to ANFH development. In this study, differentially expressed genes (DEGs) between necrosis of the femoral head (NFH) and normal samples were analyzed based on two sets of online expression profiles, GSE74089 and GSE26316. Chordin-like 2 (CHRDL2) was found to be dramatically downregulated in NFH samples. In GCs-stimulated BMECs, cellular damages were observed alongside CHRDL2 down-regulation. GCs-caused cell viability suppression, cell apoptosis promotion, tubule formation suppression, and cell migration suppression were partially abolished by CHRDL2 overexpression but amplified by CHRDL2 knockdown; consistent trends were observed in GCs-caused alterations in the protein levels of VEGFA, VEGFR2, and BMP-9 levels, and the ratios of Bax/Bcl-2 and cleaved-caspase3/Caspase3. GC stimulation significantly inhibited PI3K and Akt phosphorylation in BMECs, whereas the inhibitor effects of GCs on PI3K and Akt phosphorylation were partially attenuated by CHRDL2 overexpression but further amplified by CHRDL2 knockdown. Moreover, CHRDL2 overexpression caused improvement in GCs-induced damages to BMECs that were partially eliminated by PI3K inhibitor LY294002. In conclusion, CHRDL2 is down-regulated in NFH samples and GCs-stimulated BMECs. CHRDL2 overexpression could improve GCs-caused BMEC apoptosis and dysfunctions, possibly via the PI3K/Akt pathway.

## 1 Introduction

Steroid-induced avascular necrosis of the femoral head (ANFH) is characterized by the death of bone tissues, leading to impairment of normal reparative processes within micro-fractures in the femoral head [1]. The most predominant type of femoral head necrosis will inevitably result in a collapse of the femoral head and subsequent osteoarthritis if not treated effectively [2,3]. Most patients are indicated for total hip arthroplasty. Currently, Dexamethasone (Dex) and other glucocorticoids (GCs) are widely used to treat several disorders, including severe infection, inflammation, antishock, allergic reactions, hematologic disorders, and replacement therapy [4,5]. Due to the widespread use of Dex and other GCs, ANFH incidence has already surpassed trauma-induced necrosis of the femoral head [6]. Nevertheless, ANFH pathogenesis requires further exploration because it is critical to understand ANFH pathogenesis to develop an effective treatment regimen.

Endothelial cell damage could lead to aberrant blood coagulation and thrombus formation, whereas thrombosis and embolism may promote ANFH progression [7,8]. In dysbaric osteonecrosis, Slichter et al. [9] observed endothelial cell destruction, platelet thrombus development, and secondary fibrin deposition in the femoral head. Endothelial cell damage with a high coagulant and low fibrinolytic milieu was similarly observed by Li et al. [10], indicating the potential pathogenic processes of glucocorticoid-induced ANFH. Other studies have linked bone microvascular endothelial cells (BMECs) damage or malfunction to the development of femoral head necrosis [[11], [12], [13], [14]]. BMECs are found lining the inner layer of blood arteries and sinusoids, significantly impacting vascular homeostasis and angiogenesis [15]. Additionally, endothelial cells (ECs) underwent apoptosis in response to serum deprivation, whereas several reagents protect ECs against apoptosis to restore EC functions [16]. The amount of apoptosis is adversely connected to angiogenesis functioning and the integrity of blood vessels [17]. Furthermore, multiple investigations have concluded that chronic glucocorticoid exposure could cause regional endothelial cell malfunction, leading to apoptosis and limiting angiogenesis [18]. A few findings have been yielded on whether and how BMECs' angiogenic and apoptotic activity change during glucocorticoid-induced ANFH patients.

Growth factors and other extracellular signals activate the phosphatidylinositol-3 kinase (PI3K)/protein kinase B (Akt) pathway. PI3K activation influences various biological functions, such as cell growth, proliferation, and survival [19]. Notably, the PI3K/Akt signaling critically affected anti-apoptosis [20,21]. Wang et al. demonstrated that gastrodin activates the PI3K/Akt pathway to promote human umbilical vein endothelial cell angiogenesis [22]. Cao et al. reported that Ginkgo biloba L. extract prevents steroid-induced necrosis of the femoral head by rescuing apoptosis and dysfunction in vascular endothelial cells via the PI3K/Akt/eNOS pathway [23]. Therefore, it is hypothesized that the PI3K/Akt signaling might regulate BMECs' angiogenic and apoptotic activity. In this study, differentially expressed genes (DEGs) between NFH and normal samples were analyzed to identify critical factors affecting BMECs angiogenesis and apoptosis. The effects of the candidate gene on BMECs functions and apoptosis and the PI3K/Akt signaling were investigated. The dynamic effects of the candidate gene and PI3K inhibitor on BMECs were evaluated. Collectively, this study aims to identify key genes acting on BMEC functions and apoptosis to affect ANFH development.

## 2 Materials and methods

### 2.1 Clinical sampling

6 glucocorticoid-induced ANFH femoral head tissues were harvested from patients who suffered glucocorticoid-induced ANFH. The inclusion criteria were (1) male or female patients between 40 and 70 years old; (2) indications for total hip arthroplasty; (3) diagnosis of glucocorticoid-induced ANFH. 5 normal femoral head tissues with no internal lesions were obtained from patients undergoing artificial joint replacement due to a femoral neck fracture. Patients with ankylosing spondylitis, rheumatoid arthritis, and other inflammatory joint diseases involving the hip joint, hemophilia hip joint disease, hip tuberculosis, purulent infection, and tumors around the hip joint were excluded. The ANFH group comprised six women (average age of 37.2 ± 9.6 years) at the time of surgery, while the control group comprised five women (average age of 46.0 ± 7.7 years) at the time of surgery. The demographic characteristics of the patients and controls are listed in Table S1. All samples were treated immediately after collection in sterile conditions. The clinical sampling was performed with the approval of the Ethics Committee of Second Xiangya Hospital of Central South University (approval ID: 2022-213). Informed consent was signed by each patient enrolled.

### 2.2 Immunohistochemical (IHC) staining

The tissues were fixed, decalcified, and embedded in paraffin. The slices were subsequently deparaffinized and rehydrated through graded alcohols and washed in PBS twice for 10 min. The sections were incubated in 3 % H_2_O_2_ solution at 26 °C for 10 min to neutralize endogenous peroxidase. The antigen was repaired through a water-bath repair solution. A 40-min incubation period at 26 °C with 5 % goat serum blocked the nonspecific proteins. The slices were subsequently incubated with monoclonal primary antibody against CHRDL2 (1:100, Abcam, Cambridge, MA, USA) at 4 °C overnight. After the incubation, the slices were washed and stained with a rabbit IgG-immunohistochemical SABC kit (Boster, Wuhan, China). After a PBS washing, Hematoxylin was added for nuclei staining (0.1 % Mayer's hematoxylin). The slides were observed under a microscope after undergoing standard procedures.

### 2.3 Cell isolation and cell culture

The human bone microvascular endothelial cells were isolated as previously described [24]. Briefly, collected femoral head tissues were broken into bone grains with a rongeur. The bone grains were placed in the Dulbecco's modified Eagle's medium (DMEM) culture medium (Invitrogen, Carlsbad, CA, USA) containing type I collagenase (Thermo Fisher Scientific, Waltham, MA, USA) for digestion in a 37 °C bath for 30 min. After discarding the medium, trypsin-EDTA buffer (0.25 %; Gibco, Carlsbad, CA, USA) was added and incubated in a 37 °C bath for 10 min. The digestive suspension was collected and then centrifuged at 2000r/min for 6 min. The collected cells were subsequently purified by magnetic-activated cell sorting (MACS). Magnetic sorting was performed by a positive selection for platelet endothelial cell adhesion molecule (PECAM-1, also CD31) microBead MACS kit (Miltenyi Biotec, Bergisch Gladbach, Germany) as directed by the manufacturer. Collected BMECs were cultured in M199 medium (Gibco) supplemented with antibiotics, 10 ng/ml vascular endothelial cell growth factor, 40 U/ml heparin, and 20 % FBS (Invitrogen). The cells were cultivated at 37 °C in a humidified atmosphere containing 5 % CO_2._ The 2–4 passage cells were used for investigation. For simulating cell damages to BMECs under hormone-related ANFH, BMECs were stimulated with 0.3 mg/ml hydrocortisone (a glucocorticoid; R&D Systems, Minneapolis, MN, USA) for 24 h [25].

### 2.4 qRT-PCR assay

Femoral head tissues and BMECs were harvested, and TRIZOL (Invitrogen) was employed to extract total RNA. A reverse transcription kit (TaKaRa, Tokyo, Japan) was used to perform reverse transcription as directed by the protocol. A LightCycler 480 (Roche, Indianapolis, IN, USA) fluorescent quantitative PCR instrument was applied to detect gene expression, and the operating protocol of the fluorescent quantitative PCR kit (SYBR Green Mix, Roche Diagnostics, Indianapolis, IN) was used to set reaction conditions. A qPCR was set up with three replicates for each reaction, and GAPDH was utilized as the internal control. The amplification primers for each gene and their internal control are detailed in the sequence. The primers utilized for qRT-PCR assay are listed in Table S2.

### 2.5 MTT assay for detecting cell viability

BMECs were seeded onto 96-well plates at a concentration of approximately 1 × 10^4^ cells per well, transduced, and treated. Each well was subsequently added with 0.5 % 3–4,5‐dimethylthiazol‐2‐yl‐2,5‐diphenyl‐tetrazolium bromide (MTT), followed by incubation for 4 h at 37 °C. Subsequently, each well was added with dimethyl sulfoxide (150 μl, DMSO). The absorbance at 490 nm was measured using a spectrophotometer.

### 2.6 Flow cytometry detecting cell apoptosis

The FITC Annexin V/Dead Cell Apoptosis Kit (Invitrogen) was used to evaluate cell apoptosis. Cells were transduced, treated, and stained with 5 μL FITC Annexin V (Invitrogen) and 5 μL propidium iodide (PI) solution (100 μg/mL, Invitrogen) in 500 μL binding buffer for 30 min in light-deprived conditions. Cell apoptosis was subsequently evaluated using flow cytometry (Novocyte, USA).

### 2.7 Immunoblotting

Cells were lysed using radioimmunoprecipitation assay (RIPA) buffer (Beyotime, Shanghai, China), and total protein was harvested from the lysate. The total protein concentration was assessed using a bicinchoninic acid (BCA) kit (Beyotime). After being electrophoretically separated on a sodium dodecyl sulfate-polyacrylamide gel electrophoresis (SDS-PAGE), protein samples were transferred onto polyvinylidene fluoride (PVDF) membranes (Millipore, Billerica, MA, USA). After being blocked for 1 h with 5 % skim milk at 25 °C to prevent non-specific bindings, primary antibody incubation was performed using anti-CHRDL2 (purchased from Abcam), anti-vascular endothelial growth factor A (VEGFA), anti-vascular endothelial growth factor receptor-2 (VEGFR2), anti-bone morphogenetic protein (BMP)-9, anti-B cell lymphoma-2 (BCL-2), anti- BCL-2 associated X (Bax), anti-Caspase9/cleaved-caspase9, anti-Akt, and anti-*p*-Akt (purchased from Proteintech, Wuhan, China). Membranes were washed with tris-buffered saline containing 0.1 % Tween 20 (TBST), followed by incubation for 1 h at 37 °C with proper secondary antibodies. Finally, the membranes were incubated with ECL western blotting substrate kit (Abcam) and observed by a chemiluminescent imaging system (Tanon-5200, Tanon, Beijing, China)

### 2.8 Cell transduction

The pLVX-shRNA2 vector containing the short hairpin RNA targeting CHRDL2 (sh-CHRDL2-1/2; constructed by Ori-Bio Ltd. co, Changsha, China) was transduced into cells to achieve CHRDL2 knockdown. pcDNA3.1 contained CHRDL2 mRNA (CHRDL2; constructed by Ori-Bio) and was transduced into cells to achieve CHRDL2 overexpression. Cells were transfected using the Lipofectamine 3000 reagent (Invitrogen) as directed by the manufacturer. The transfection media was discarded 6 h following transfection. Cells were further cultured for 48 h. The sequences of overexpressing vectors and shRNA vectors are listed in Table S2.

### 2.9 Tubule formation assay

A 24-well plate was pre-coated with Corining Matrigel Matrix (300 μL). The cells were subsequently suspended by a complete culture medium at 4 × 10^5^/ml concentration and added to the 24-well. Following incubation for 6 h in a 5 % CO_2_ atmosphere at 37 °C, the branch nodes formed by BMECs were observed and counted under an optical microscope in five randomly selected visual areas.

### 2.10 Wound healing assay detecting cell migration

Pipette tips were used to scrape the BMECs cultivated into six-well plates, and the ablated cells were rinsed away with PBS. At 0, 24, and 48 h after wounding, cells were photographed in the same area of the culture plate. The cell mobility was assessed by measuring the wound breadth following a previously reported formula [26].

### 2.11 Statistical analysis

The statistical tool SPSS version 21.0 (SPSS Inc., New York, NY, USA) was used for all statistical analyses. A one-way analysis of variance (ANOVA) followed by Tukey's post‐hoc test was applied to analyze three groups or more. A student's *t*-test was applied to contrast the differences between the two experimental groups. The significance level was set at *P* < 0.05.

## 3 Results

### 3.1 Selecting genes playing a critical role in ANFH

**Fig. 1:**
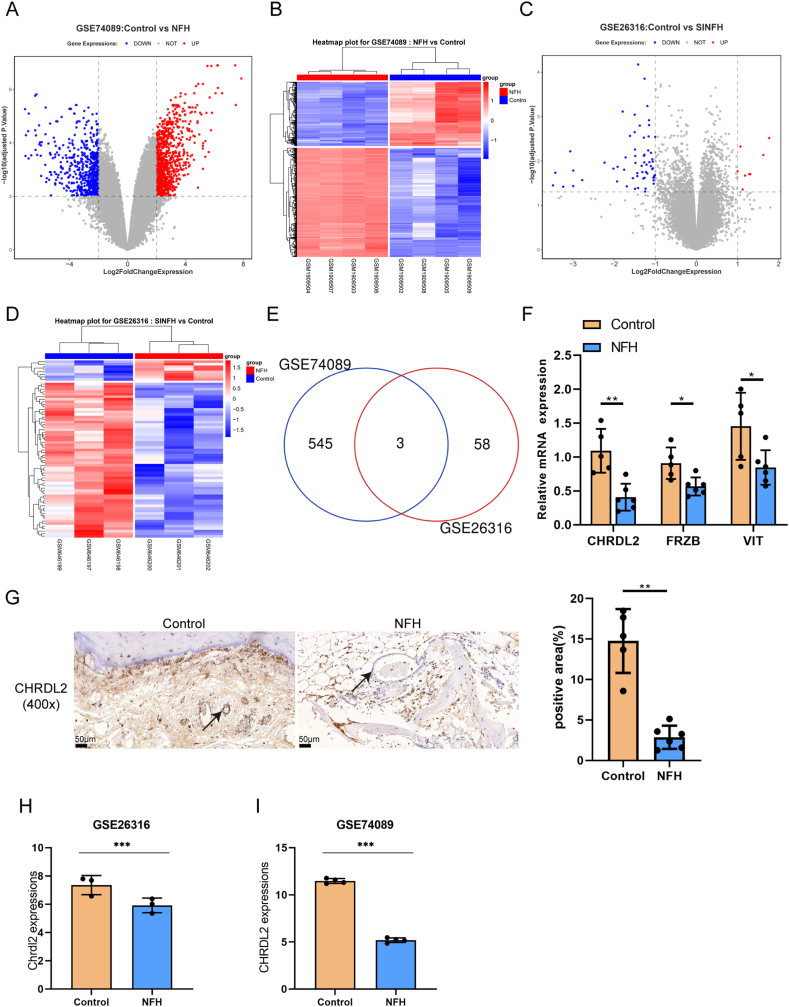
Selecting genes playing a critical role in avascular necrosis of the femoral head (A–B) GSE74089 contains gene expression profiling of 12 patients with necrosis of the femoral head (NFH) and 12 healthy controls. Differentially expressed genes were shown in volcano plots and hierarchical clustering heatmap. (C–D) GSE26316 contains a gene expression profile of rats with steroid-induced necrosis of the femoral head and normal control rats. Differentially expressed genes were shown in volcano plots and hierarchical clustering heatmap. (E) Down-regulated genes in NFH samples were compared, and overlapped genes were shown. (F) VIT, FRZB and CHRDL2 mRNA levels were detected in collected ANFH and normal control femoral head tissue samples using qRT-PCR assay. (G) CHRDL2 protein levels were evaluated in collected ANFH and normal control femoral head tissue samples using Immunohistochemical staining (IHC). The black arrows indicate vascular endothelial cells. Scale bar = 50 μm. (H–I) CHRDL2 expression in NFH and normal control samples according to GSE74089 and GSE26316. *P < 0.05, **P < 0.01, ***P < 0.001 compared to control group.

The first step in identifying key genes involved in the formation of ANFH was to compare and select DEGs between ANFH and normal samples. GSE74089 contains gene expression profiling of 12 patients with necrosis of the femoral head (NFH) and 12 healthy controls (Fig. 1A and B); DEGs were identified using the Microarray Significance Analysis (SAM) software. An expression matrix of 21754 genes was yielded by 34183 probes, and 548 decreased genes (logFC < −2, *P* < 0.05) and 932 increased genes (logFC >2, *P* < 0.05) were yielded by the limma package of R language (Fig. 1A and B). GSE26316 contains the gene expression profile of rats with steroid-induced necrosis of femoral head and normal control rats (Fig. 1C and D); an expression matrix of 15247 rat genes were yielded by 31042 probes, and 61 decreased genes (logFC < −1, *P* < 0.05) and 8 increased genes (logFC >1, *P* < 0.05) were yielded by the limma package of R language (Fig. 1C and D). Down-regulated DEGs were then compared, and vitrin (VIT), frizzled-related protein (FRZB), and CHRDL2 were down-regulated in NFH samples (Fig. 1E), CHRDL2 was more down-regulated (Table 1). Moreover, VIT, FRZB, and CHRDL2 mRNA levels were detected in the femoral head tissues of ANFH and normal samples. When compared to normal samples, VIT, FRZB, and CHRDL2 mRNA levels were downregulated in ANFH samples. Among them, CHRDL2 had the lowest expression level (Fig. 1F). Furthermore, CHRDL2 protein levels were evaluated in collected ANFH and normal samples using IHC staining; consistently, CHRDL2 protein levels showed to be remarkably reduced in ANFH samples than normal control (Fig. 1G). Also, CHRDL2 expression in NFH and normal control samples according to GSE74089 and GSE26316 was shown in Fig. 1H and I. CHRDL2 expression was decreased in NFH samples.

**Table 1:** Expression of overlapped down-regulated genes in avascular necrosis of the femoral head samples according to GSE74089 and GSE26316.

Gene	logFC	AveExpr	t	P.Value	adj.P.Val	B
CHRDL2	−6.28604	8.32085	−34.255	1.13E-09	1.54E-06	12.74343
VIT	−2.46903	8.440103	−17.3302	1.98E-07	2.88E-05	8.028863
FRZB	−5.96496	9.644007	−6.67718	0.000189	0.001733	0.724987

### 3.2 CHRDL2 effects on GCs-induced damages to BMECs

**Fig. 2:**
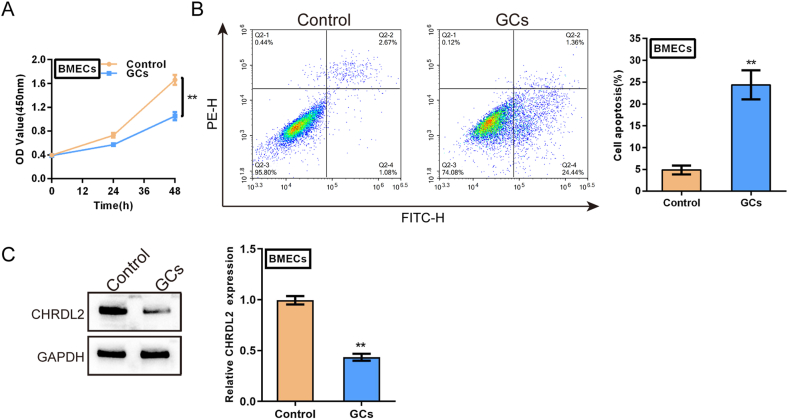
Damages to bone microvascular endothelial cells (BMECs) by glucocorticoids (GCs) treatment (A–C) BMECs were stimulated with 0.3 mg/ml GCs for 24 h and examined for (A) cell viability using MTT assay; (B) cell apoptosis using Flow cytometry; (C) the protein levels of CHRDL2 using Immunoblotting. The uncropped immunoblotting images of Fig. 2(C) were shown in Supplementary materials. **P < 0.01 compared to control group.

BMECs were stimulated with 0.3 mg/ml GCs for 24 h and examined for cell phenotypes to simulate damage to BMECs during hormone-related ANFH. It can be inferred from Fig. 2A and B that GC stimulation markedly suppressed cell viability but enhanced cell apoptosis, indicating GCs-caused cell damage to BMECs. Moreover, GCs stimulation dramatically reduced CHRDL2 proteins (Fig. 2C).

**Fig. 3:**
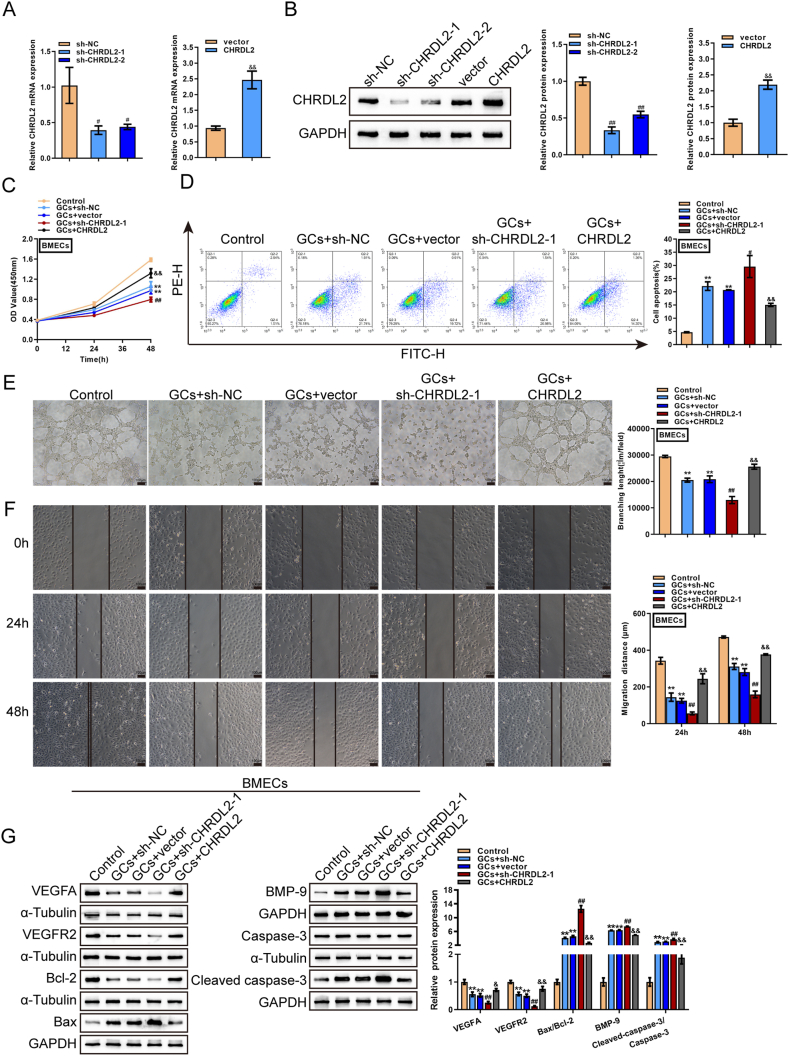
CHRDL2 effects on GCs-induced damages to BMECs (A–B) CHRDL2 knockdown or overexpression was achieved in BMECs by transducing short hairpin RNA targeting CHRDL2 (sh-CHRDL2-1/2) or plasmid overexpressing CHRDL2 (CHRDL2); CHRDL2 knockdown or overexpression was confirmed using (A) qRT-PCR or (B) Western blot assays. #*P* < 0.05, ##*P* < 0.01 compared to sh-NC group; && *P* < 0.01 compared to vector group.(C–G) BMECs were transduced with sh-CHRDL2-1 or CHRDL2, stimulated with 0.3 mg/ml GCs for 24 h, and examined for (C) cell viability using MTT assay; (D) cell apoptosis using Flow cytometry; (E) tubule formation by tubule formation assay; (F) cell migration using Wound healing assay; (G) the protein levels of VEGFA, VEGFR2, BMP-9, Bcl-2, Bax, Caspase3 and cleaved-caspase3 using Immunoblotting. The uncropped immunoblotting images of Fig. 3(B–G) were shown in Supplementary materials. **P < 0.01 compared to control group; #*P* < 0.05, ##*P* < 0.01 compared to GCs + sh-NC group; & *P* < 0.05, && *P* < 0.01 compared to GCs + vector group.

Considering CHRDL2 down-regulation in GCs-stimulated BMECs, CHRDL2 knockdown or overexpression was achieved in BMECs by transducing sh-CHRDL2-1/2 or CHRDL2 vector; CHRDL2 knockdown or overexpression was confirmed through qRT-PCR (Fig. 3A) and Western blot (Fig. 3B). sh-CHRDL2-1 was used in subsequent investigations due to superior efficiency. BMECs were subsequently transduced with sh-CHRDL2-1 or CHRDL2, stimulated with 0.3 mg/ml GCs for 24 h, and examined for cell phenotypes. GCs-caused cell viability suppression (Fig. 3C), cell apoptosis promotion (Fig. 3D), tubule formation suppression (Fig. 3E), and cell migration suppression (Fig. 3F) were partially abolished by CHRDL2 overexpression but amplified by CHRDL2 knockdown (Fig. 3C–F). Regarding related markers, GCs stimulation decreased the protein levels of VEGFA and VEGFR2 but increased BMP-9 levels and the ratios of Bax/Bcl-2 and cleaved-caspase3/Caspase3 (Fig. 3G); GCs-induced alterations in these factors were partially eliminated by CHRDL2 overexpression but amplified by CHRDL2 knockdown (Fig. 3G). These findings suggest that CHRDL2 overexpression could partially improve GCs-induced damages to BMECs, but CHRDL2 knockdown further amplified the damages.

### 3.3 PI3K/Akt signaling mediates CHRDL2 functions on GCs-induced damages to BMECs

**Fig. 4:**
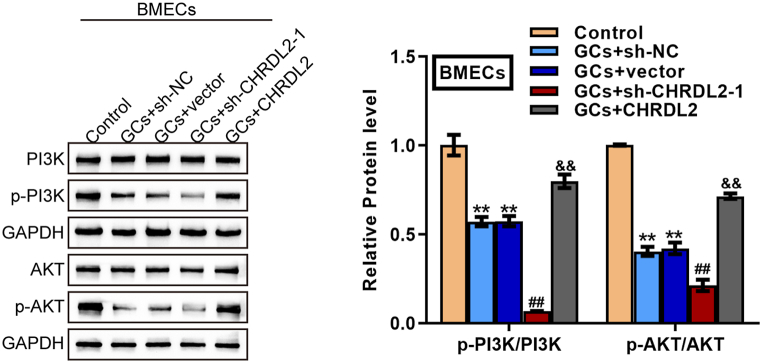
CHRDL2 effects on the Akt signaling in GCs-treated BMECs BMECs were transduced with sh-CHRDL2-1 or CHRDL2, stimulated with 0.3 mg/ml GCs for 24 h, and examined for the protein levels of PI3K, p-PI3K, Akt and *p*-Akt using Immunoblotting. The uncropped immunoblotting images of Fig. 4 were shown in Supplementary materials. **P < 0.01 compared to control group; ##*P* < 0.01 compared to GCs + sh-NC group; && *P* < 0.01 compared to GCs + vector group.

**Fig. 5:**
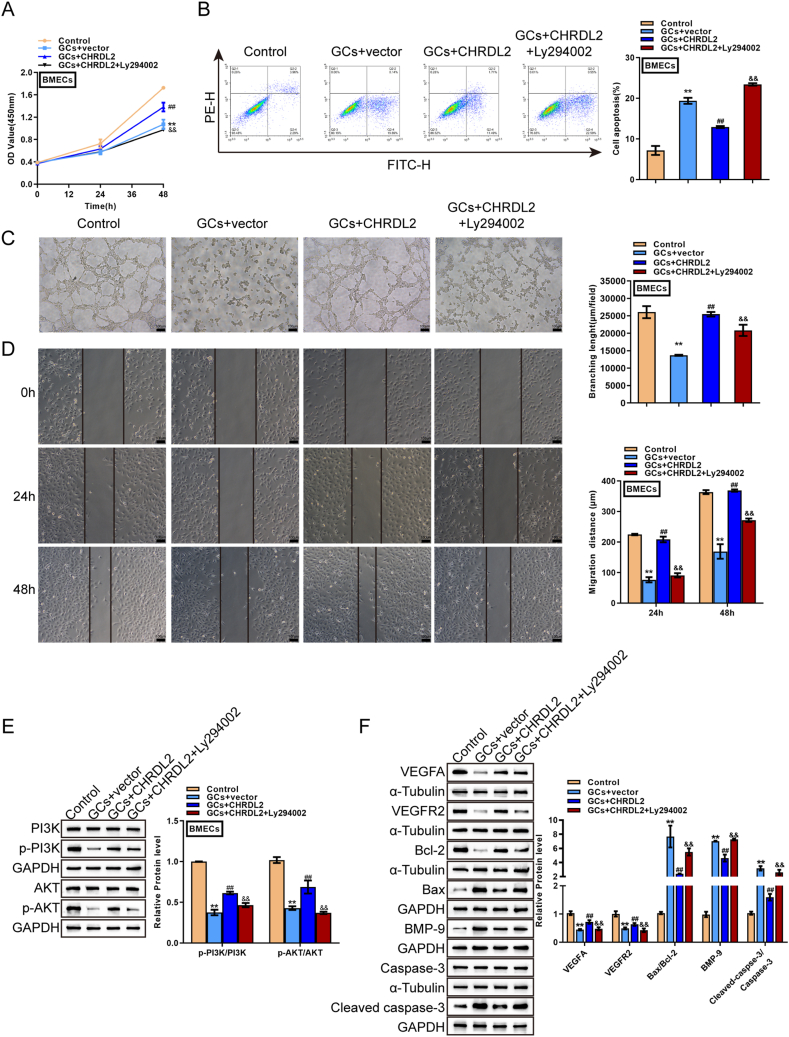
PI3K/Akt signaling mediates CHRDL2 functions on GCs-induced damages to BMECs (A–F) BMECs were transduced with CHRDL2 overexpression vector, stimulated with 0.3 mg/ml GCs for 24 h in the presence or absence of LY294002, and examined for (A) cell viability using MTT assay; (B) cell apoptosis using Flow cytometry; (B) tubule formation by tubule formation assay; (D) cell migration using wound healing assay; (E) the protein levels of PI3K, p-PI3K, Akt and *p*-Akt using Immunoblotting; (F) the protein levels of CHRDL2, VEGFA, VEGFR2, BMP-9, Bcl-2, Bax, Caspase3, and cleaved-caspase3 using Immunoblotting. The uncropped immunoblotting images of Fig. 5(E and F) were shown in Supplementary materials. **P < 0.01 compared to control group; ##*P* < 0.01 compared to GCs + vector group; && *P* < 0.01 compared to GCs + CHRDL2 group.

As mentioned above, the PI3K/Akt signaling critically affected anti-apoptosis [27,28]. The role of the PI3K/Akt signaling in CHRDL2 functions on GCs-induced BMEC damages was subsequently investigated. BMECs were transduced with sh-CHRDL2-1 or CHRDL2, stimulated with 0.3 mg/ml GCs for 24 h, and examined for the protein levels of PI3K, p-PI3K, Akt, and *p*-Akt. Fig. 4 shows that GC stimulation dramatically reduced p-PI3K and *p*-Akt proteins; the inhibition effect of GCs on PI3K and Akt phosphorylation was partially attenuated by CHRDL2 overexpression but was further amplified by CHRDL2 knockdown. BMECs were then transduced with CHRDL2, stimulated with 0.3 mg/ml GCs for 24 h with or without the PI3K inhibitor LY294002, and examined for cell phenotypes, the alterations in the PI3K/Akt pathway, and related markers. Regarding cell phenotypes, GCs-caused cell viability suppression (Fig. 5A), cell apoptosis promotion (Fig. 5B), tubule formation suppression (Fig. 5C), and cell migration suppression (Fig. 5D) were partially abolished by CHRDL2 overexpression (Fig. 5A–F); however, when co-treated with LY294002, CHRDL2 overexpression caused improvement of GCs-caused cell damage was partially attenuated by LY294002 (Fig. 5A–D). Regarding the PI3K/Akt signaling, GCs stimulation inhibited, whereas CHRDL2 overexpression promoted PI3K and Akt phosphorylation; the effects of CHRDL2 overexpression on PI3K and Akt phosphorylation were partially eliminated by LY294002 (Fig. 5E). Consistently, GCs-induced alterations in VEGFA, VEGFR2, and BMP-9 protein levels and the ratios of Bax/Bcl-2 and cleaved-caspase3/Caspase3 were partially eliminated by CHRDL2 overexpression; however, LY294002 treatment attenuated CHRDL2 overexpression effects on GCs-induced alterations in these factors (Fig. 5F). In summary, the PI3K/Akt pathway might mediate the effects of CHRDL2 upon GCs-caused BMECs damages.

## 4 Discussion

In this study, DEGs between NFH and normal samples were analyzed based on two sets of online expression profiles, GSE74089 and GSE26316, and CHRDL2 was found to be dramatically downregulated in NFH samples. In GCs-stimulated BMECs, cellular damage was observed alongside CHRDL2 down-regulation. GCs-caused cell viability suppression, cell apoptosis promotion, tubule formation suppression, and cell migration suppression were partially abolished by CHRDL2 overexpression but amplified by CHRDL2 knockdown; consistent trends were observed in GCs-caused alterations in the protein levels of VEGFA, VEGFR2, and BMP-9 levels, and the ratios of Bax/Bcl-2 and cleaved-caspase3/Caspase3. GC stimulation significantly inhibited PI3K and Akt phosphorylation in BMECs, but the inhibition effect of GCs on PI3K and Akt phosphorylation was partially attenuated by CHRDL2 overexpression but further amplified by CHRDL2 knockdown. Moreover, CHRDL2 overexpression caused improvements in GCs-induced damage against BMECs that were partially eliminated by the PI3K inhibitor LY294002.

Previously, CHRDL2 has been recognized as an oncogenic factor in breast cancer, lung cancer, colon cancer, and osteosarcoma, with elevated levels in tissue samples and promoting tumorigenesis [[29], [30], [31]]. In osteosarcoma, CHRDL2 knockdown inhibited colony formation capacity and the abilities of osteosarcoma cells to proliferate, migrate, and invade *in vitro* [31]. As mentioned above, the amount of apoptosis is adversely connected to the functioning of angiogenesis and the integrity of blood vessels [32]. Moreover, chronic glucocorticoid exposure might cause regional endothelial cell malfunction, leading to cell death and limiting angiogenesis [18]. According to online databases and experimental studies, CHRDL2 expression was shown to be down-regulated in ANFH samples and GCs-stimulated BMECs in this study, suggesting its possible significance in influencing BMEC apoptosis and angiogenesis. GCs have long been reported to impair endothelial cell functions. For example, GC stimulation in young mice elicits the senescence of vascular endothelial cells in long bone metaphysis, and suppression of such senescence promotes GCs-impaired osteogenesis-angiogenesis coupling [33]. In this study, GC stimulation suppressed BMECs proliferation, tubule formation, and migration but promoted cell apoptosis, whereas CHRDL2 overexpression significantly attenuated GCs-caused damages to BMECs by promoting cell proliferation, tubule formation and migration but inhibiting cell apoptosis. These findings indicate that CHRDL2 overexpression partially improves BMEC apoptosis and dysfunctions caused by GCs.

CHRDL2 is a BMP antagonist blocking BMPs from engaging with their corresponding cell surface receptors [34]. BMPs, particularly BMP-9, directly impact EC behavior and modulate the effects of other factors, including VEGF, on endothelial physiology [35]. Reportedly, BMP-9 inhibited EC proliferation and angiogenesis. For example, according to Yoshimatsu et al. [36], a BMP-9 treatment regimen lasting 48 h could account for 65 % of the decrease in HDLEC proliferation, indicating the suppressive effect of BMP-9 upon new lymphatic vessel development. Blocking BMP-9 using recombinant human ALK-1 extracellular domain/Fc (ALK-1ecd) promotes HMVEC cell growth [37]. Furthermore, BMP-9 can affect ECs' ability to form new vasculature after VEGF therapy [35,38]. In *ex vivo* mouse fetal models, 7-day parallel stimulations with BMP-9 and VEGF reveal that BMP-9 suppresses vascularization [35]. In this study, consistent alterations in BMP-9 and VEGFA were observed, whereby CHRDL2 overexpression elevated the protein levels of VEGFA and VEGFR2, decreased BMP-9 levels, and decreased the ratios of Bax/Bcl-2 and cleaved-caspase3/Caspase3 in GCs-stimulated BMECs, suggesting that CHRDL2 affects BMECs apoptosis and angiogenesis through BMP-related manners.

The PI3K/Akt pathway has been reported to participate in endothelial cell apoptosis and functions [[39], [40], [41]]. Notably, CHRDL2 has been reported to promote the proliferation and metastasis of osteosarcoma cells through the BMP‐9/PI3K/Akt signaling pathway; by binding with BMP‐9, CHRDL2 decreased BMP‐9 expression [31]. Taking into account that BMP-9 binds to receptor ALK1, which may inhibit PI3K/Akt signaling [31], this study also examined the involvement of the PI3K/Akt signaling in CHRDL2 functions upon BMECs. Consistent with previous studies, inhibited PI3K and Akt phosphorylation was observed with GCs-caused BMEC dysfunctions. Moreover, PI3K inhibitor LY294002 amplified GCs-caused damage to BMECs, and CHRDL2 overexpression caused improvement in BMECs apoptosis and angiogenesis were partially eliminated when BMECs were treated with PI3K inhibitor LY294002. The PI3K/Akt pathway might mediate the improving effects of CHRDL2 overexpression on GC-caused BMEC apoptosis and dysfunction.

However, the limitations of this study remain to be considered. Firstly, disrupting upstream translation in mRNAs is associated with disease [42], while the upstream regulation mechanism of CHRDL2 remains unclear. Furthermore, all of our investigations were conducted in cells. The findings probably cannot be directly applied to people. In further research, rodent or mouse models could be used in investigations.

In conclusion, CHRDL2 is down-regulated in NFH samples and GCs-stimulated BMECs. CHRDL2 overexpression could improve GCs-caused BMECs apoptosis and dysfunctions, possibly through the PI3K/Akt pathway.

## Ethics and consent

The clinical sampling was performed with the approval of the Ethics Committee of Second Xiangya Hospital of Central South University (approval ID: 2022-213) on November 2nd, 2022. Written informed consent was signed by each patient enrolled.

## Funding

This study was supported by the 10.13039/501100001809National Natural Science Foundation of China (no. 82300357) and the 10.13039/501100004735Natural Science Foundation of Hunan Province, China (no. 2021JJ40834).

## Data availability statement

The datasets GSE74089 and GSE26316 uesd in this study can be found in online Gene Expression Omnibus (GEO) data repository (http://www.ncbi.nlm.nih.gov/geo).

## CRediT authorship contribution statement

**Xianzhe Huang:** Writing – original draft, Visualization, Investigation, Formal analysis. **Shuo Jie:** Resources, Investigation, Formal analysis. **Wenzhao Li:** Resources, Investigation, Formal analysis. **Chan Liu:** Writing – review & editing, Supervision, Project administration, Investigation, Conceptualization.

## Declaration of competing interest

The authors declare that they have no known competing financial interests or personal relationships that could have appeared to influence the work reported in this paper.
