# Supplementary material for: CHRDL2 activates the PI3K/AKT pathway to ameliorate glucocorticoid-induced damages to bone microvascular endothelial cells (BMECs)
Source: Heliyon. 2024 Jun 28;10(13):e33867. doi: 10.1016/j.heliyon.2024.e33867 (PMC11268171; doi:10.1016/j.heliyon.2024.e33867)

1. Uncropped blots for Figure 2.


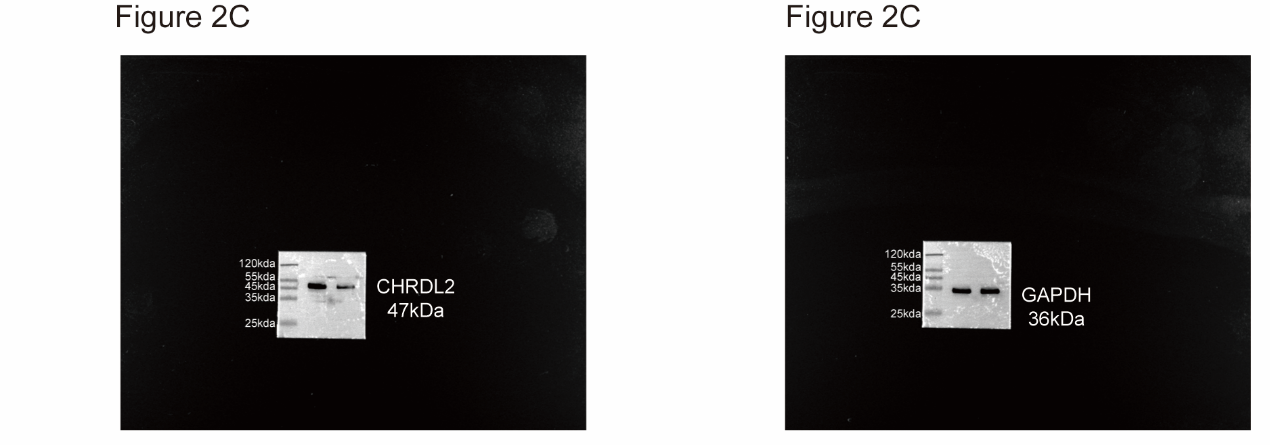


1. Uncropped blots for Figure 3.


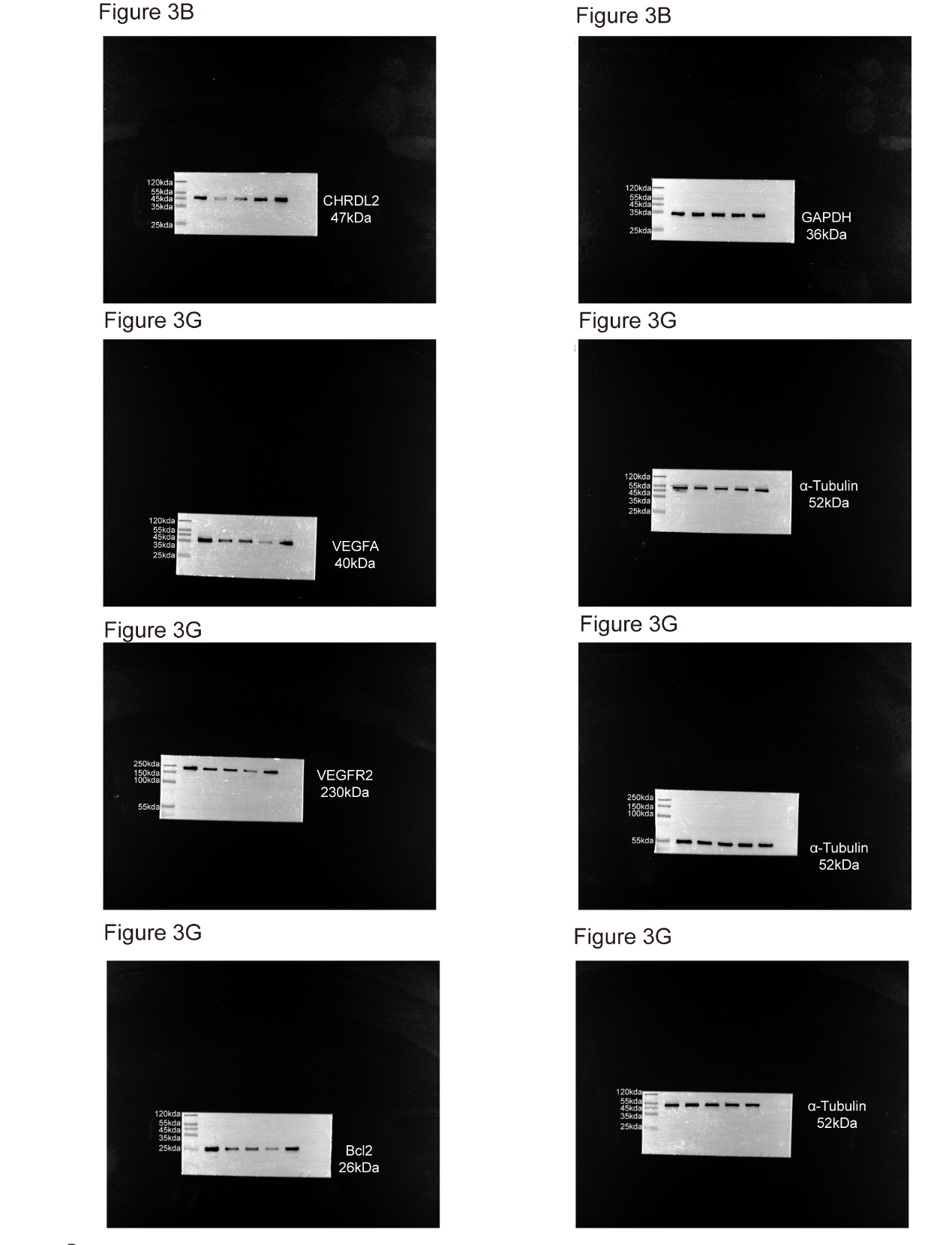


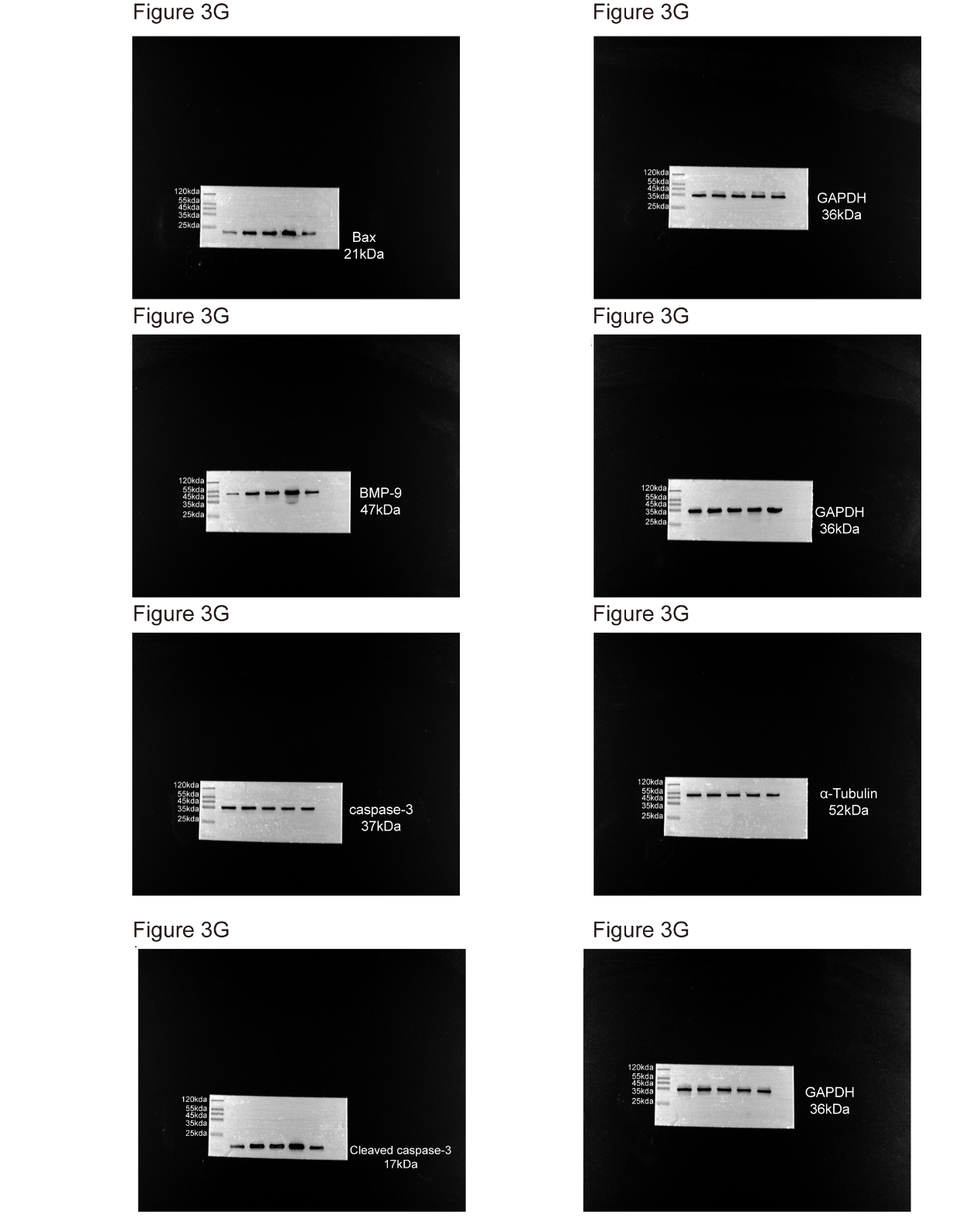


1. Uncropped blots for Figure 4.


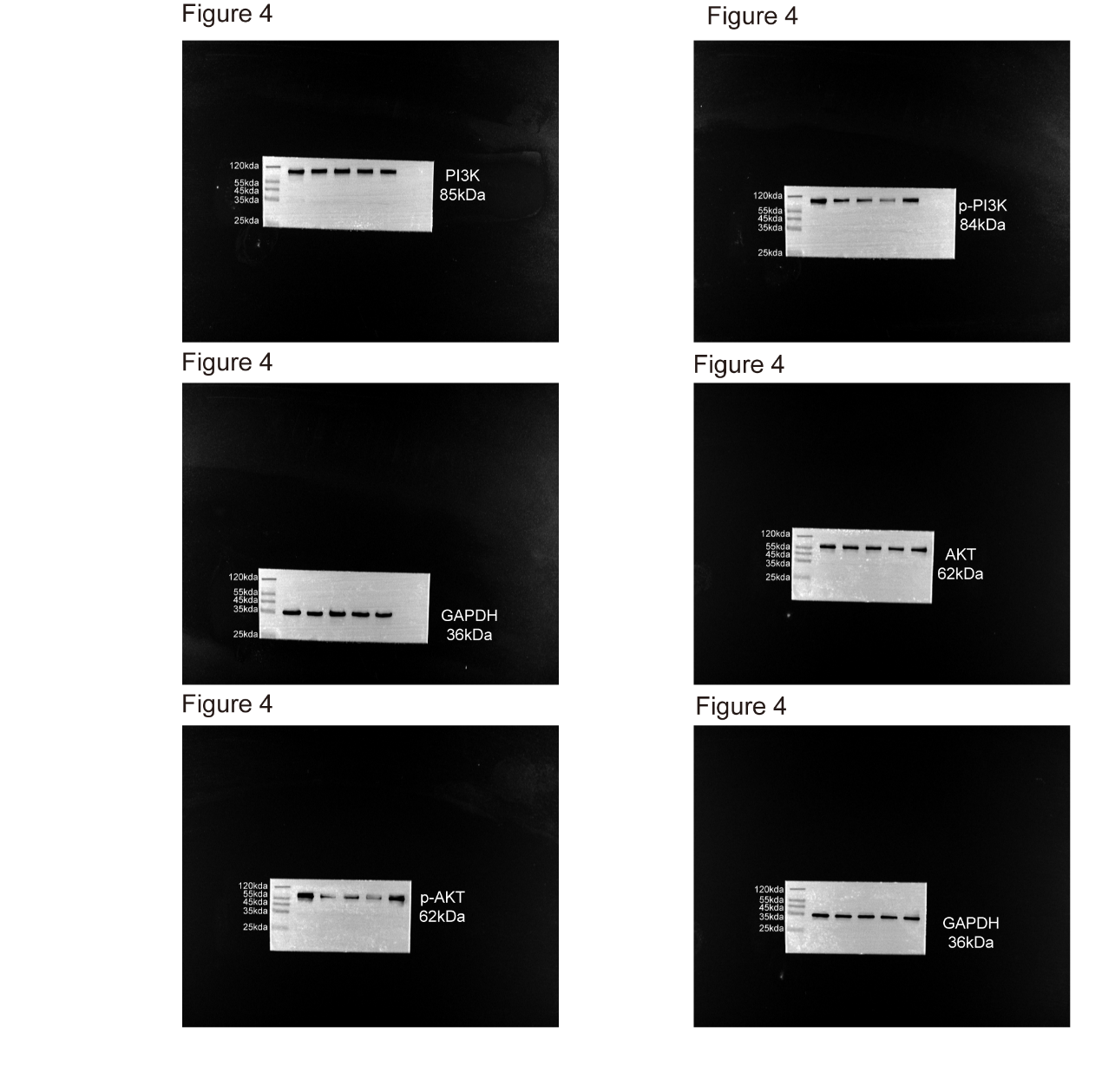


1. Uncropped blots for Figure 5.


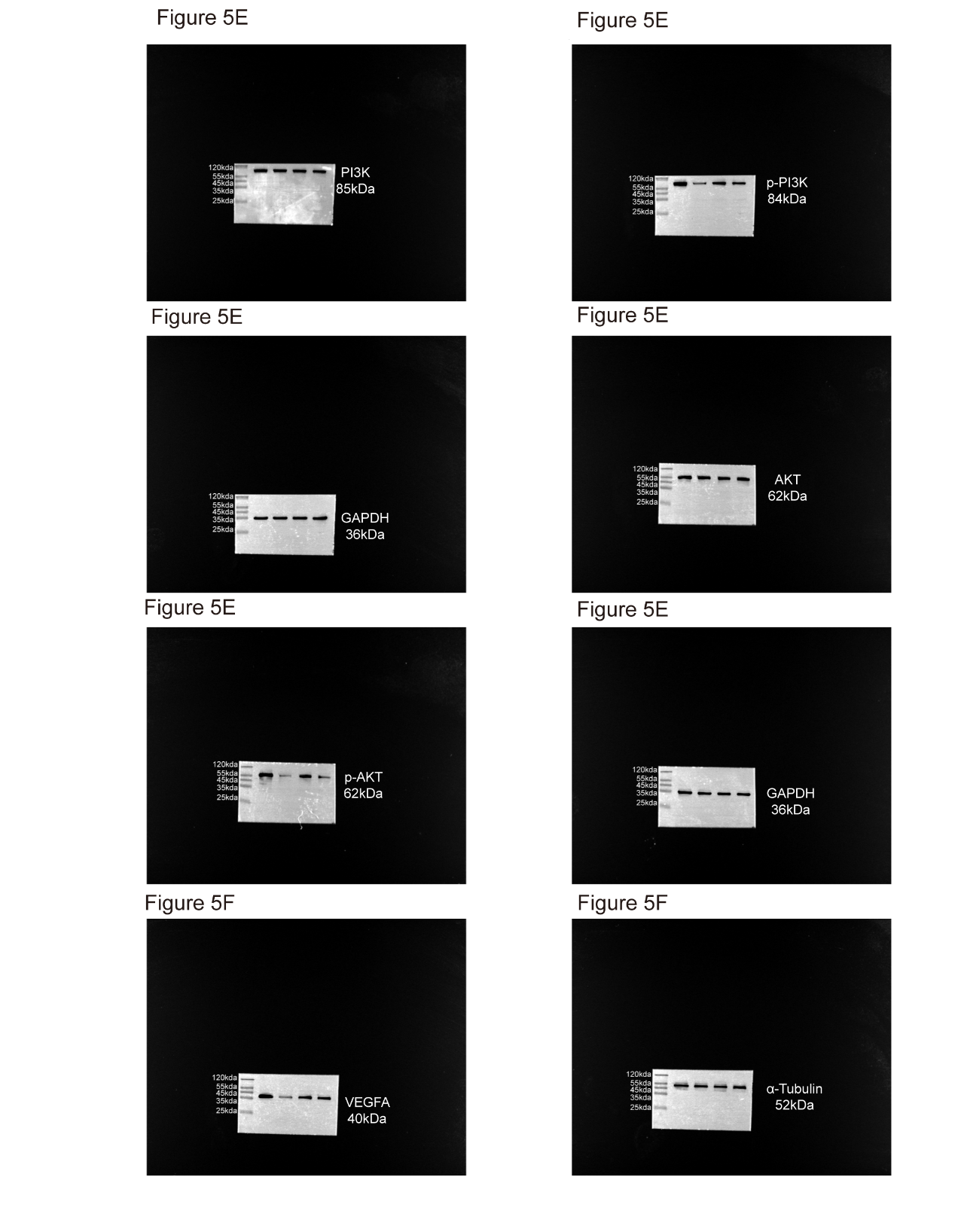

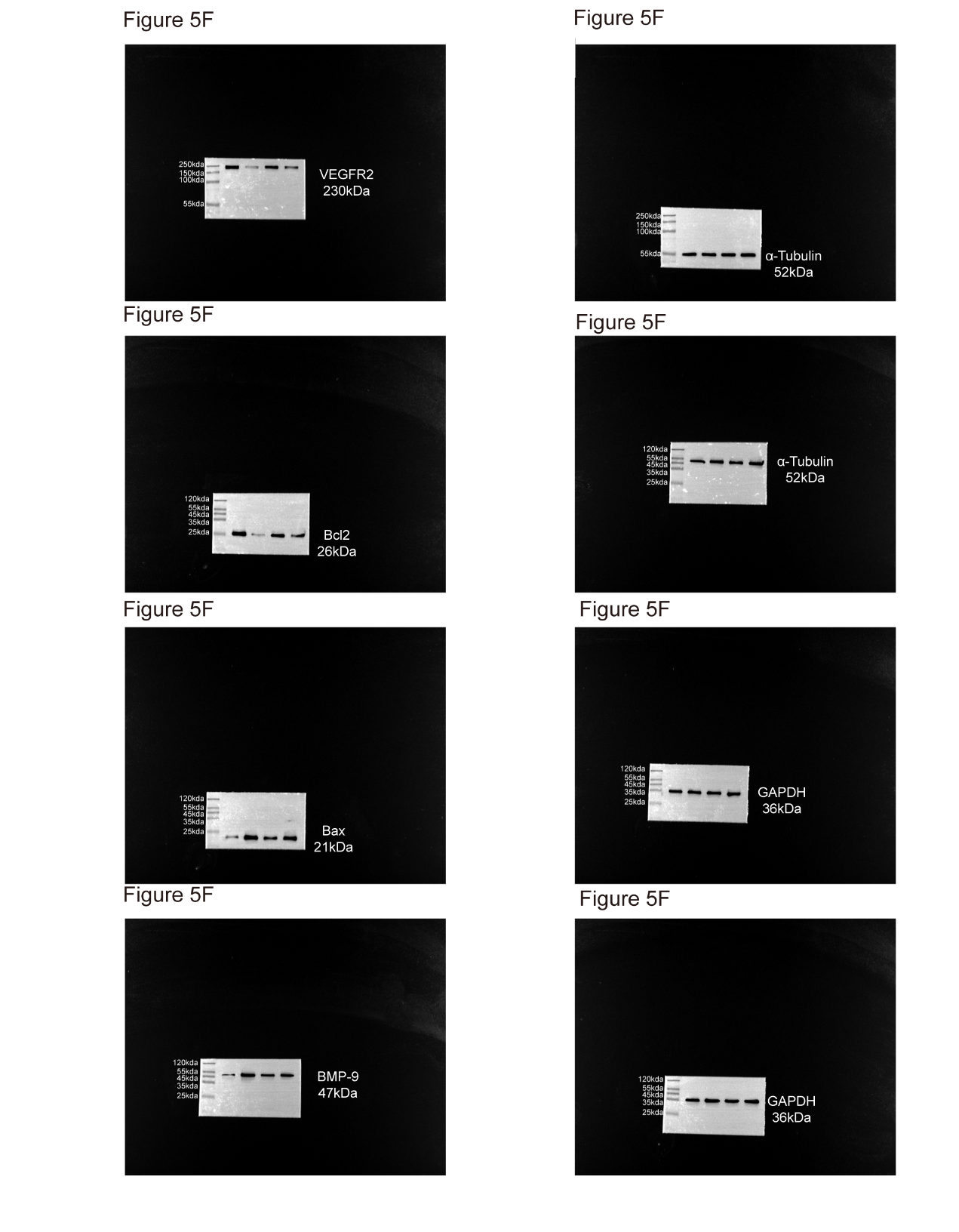

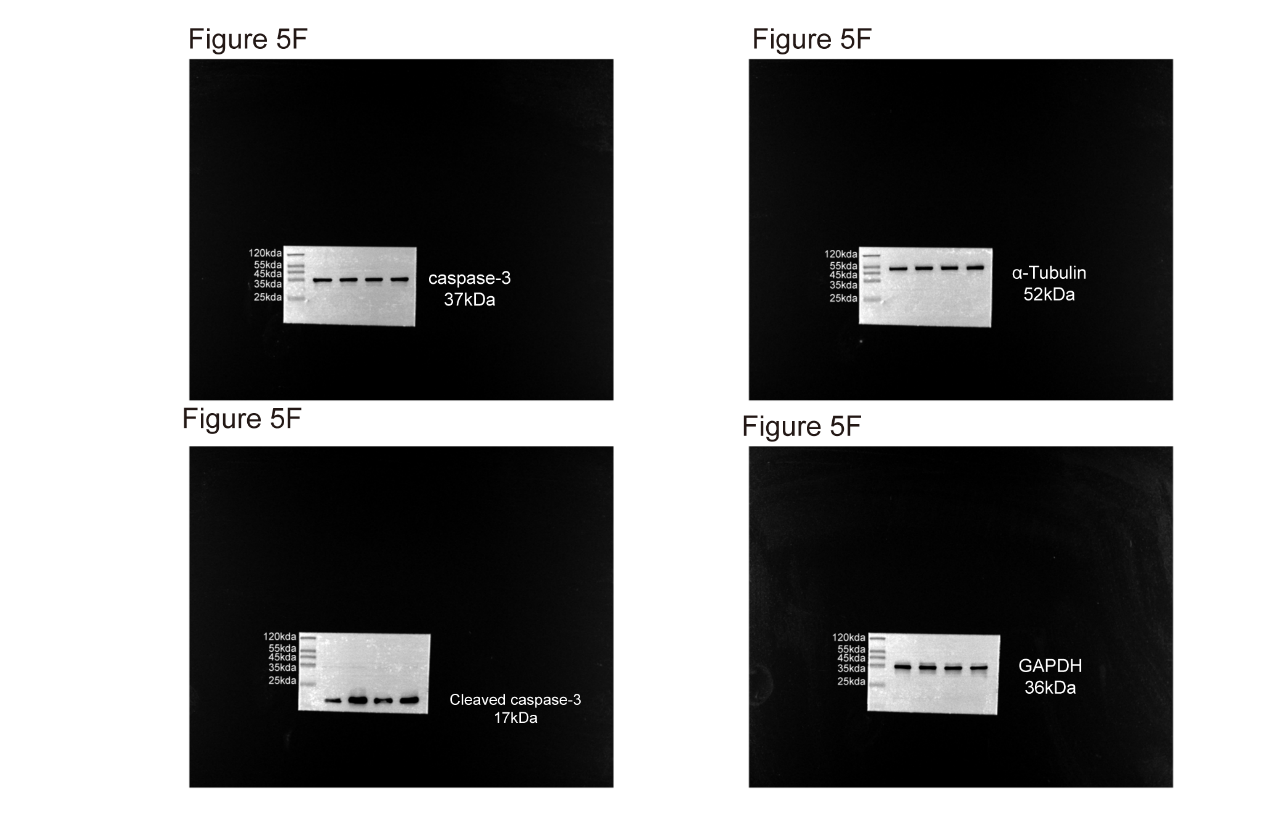

Supplement: Multimedia component 1 [file mmc1.docx]
