# Supplementary material for: CHRDL2 activates the PI3K/AKT pathway to ameliorate glucocorticoid-induced damages to bone microvascular endothelial cells (BMECs)
Source: Heliyon. 2024 Jun 28;10(13):e33867. doi: 10.1016/j.heliyon.2024.e33867 (PMC11268171; doi:10.1016/j.heliyon.2024.e33867)
Supplement: Multimedia component 2 [file mmc2.docx]

**Table S1. Demographic characteristics of the patients and controls**

| Number | Sex | Age | Height  (cm) | Weight  (kg) | Diagnosis | Stage | Underlying Disease | Duration of the usage of glucocorticoid (days) |
| --- | --- | --- | --- | --- | --- | --- | --- | --- |
| ONFH_1 | F | 55 | 163 | 62 | Steroid-induced ONFH | ARCOⅢ | SLE | 28 |
| ONFH_2 | F | 40 | 160 | 55 | Steroid-induced ONFH | ARCOIV | SLE | 30 |
| ONFH_3 | F | 32 | 164 | 56 | Steroid-induced ONFH | ARCOIV | SLE | 19 |
| ONFH_4 | F | 33 | 155 | 63 | Steroid-induced ONFH | ARCOⅢ | SLE | 25 |
| ONFH_5 | F | 35 | 163 | 65 | Steroid-induced ONFH | ARCOⅢ | NS | 40 |
| ONFH_6 | F | 28 | 158 | 70 | Steroid-induced ONFH | ARCOⅢ | NS | 36 |
| Normal_1 | F | 42 | 153 | 57 | DDH (bilateral hip) | Crowe Ⅰ | - | - |
| Normal_2 | F | 48 | 157 | 56 | DDH (bilateral hip) | Crowe Ⅰ | - | - |
| Normal_3 | F | 35 | 150 | 62 | DDH (bilateral hip) | Crowe IV | - | - |
| Normal_4 | F | 50 | 162 | 66 | DDH (bilateral hip) | Crowe Ⅱ | - | - |
| Normal_5 | F | 55 | 163 | 60 | DDH (bilateral hip) | Crowe Ⅱ | - | - |

NoteS: ONFH: osteonecrosis of the femoral head; F: female; DDH: developmental dysplasia of the hip; ARCO: Association ResearchCirculation Osseous; NS: Nephrotic Syndrome; SLE: systemic lupus erythematosus.
