# Supplementary material for: CHRDL2 activates the PI3K/AKT pathway to ameliorate glucocorticoid-induced damages to bone microvascular endothelial cells (BMECs)
Source: Heliyon. 2024 Jun 28;10(13):e33867. doi: 10.1016/j.heliyon.2024.e33867 (PMC11268171; doi:10.1016/j.heliyon.2024.e33867)
Supplement: Multimedia component 3 [file mmc3.docx]

**Table S2. The qRT-PCR primers and vector construction primers in the study**

|  | **Gene** | **Primer** | **Sequence (5’-3’)** |
| --- | --- | --- | --- |
| **PCR primers** | VIT | Forward | ATACTTGTTCGGAAGGTTGCTG |
|  |  | Reverse | CGTGGTAGGGATAACGATTGGA |
|  | FRZB | Forward | CAGTGAACGCTGTAAATGTAAG |
|  |  | Reverse | TTTAGCCCGAATGACATAGTTG |
|  | CHRDL2 | Forward | GCAAAGATGAGGCAAGTGAGCAAT |
|  |  | Reverse | GGGTCTGAAGTGGCGAGGGATG |
|  | GAPDH | Forward | ACAGCCTCAAGATCATCAGC |
|  |  | Reverse | GGTCATGAGTCCTTCCACGAT |
| **Vector construction primers** | sh-NC | Forward | GATCCGCAGATGAAGGCACGGTCACGCTCGAGCGTGACCGTGCCTTCATCTGCTTTTTG |
|  |  | Reverse | AATTCAAAAAGCAGATGAAGGCACGGTCACGCTCGAGCGTGACCGTGCCTTCATCTGCG |
|  | sh-CHRDL2-1 | Forward | GATCCGGAAGCAAGACTTCCAGAAAGCTCGAGCTTTCTGGAAGTCTTGCTTCCTTTTTG |
|  |  | Reverse | AATTCAAAAAGGAAGCAAGACTTCCAGAAAGCTCGAGCTTTCTGGAAGTCTTGCTTCCG |
|  | sh-CHRDL2-2 | Forward | GATCCGACAAAGTGACCAAGACATAACTCGAGTTATGTCTTGGTCACTTTGTCTTTTTG |
|  |  | Reverse | AATTCAAAAAGACAAAGTGACCAAGACATAACTCGAGTTATGTCTTGGTCACTTTGTCG |
|  | Vector | Forward | / |
|  |  | Reverse | / |
|  | CHRDL2 | Forward | CTAGCGTTTAAACTTAAGCTTATGGTTCCCGAGGTGAGGG |
|  |  | Reverse | TGCTGGATATCTGCAGAATTCTTAGGTCTTTGTTATGTCTTGGTCACT |
